# Growth of organic crystals via attachment and transformation of nanoscopic precursors

**DOI:** 10.1038/ncomms15933

**Published:** 2017-06-21

**Authors:** Yuan Jiang, Matthias Kellermeier, Denis Gebauer, Zihao Lu, Rose Rosenberg, Adrian Moise, Michael Przybylski, Helmut Cölfen

**Affiliations:** 1 Research Institute for Biomimetics and Soft Matter, College of Materials, Xiamen University, Si Ming Nan Lu 422, 361005 Xiamen, China; 2 Physical Chemistry, University of Konstanz, Universitätsstraße 10, 78457 Konstanz, Germany; 3 Colloid Chemistry, Max-Planck-Institute of Colloids and Interfaces, Research Park Golm, 14424 Potsdam, Germany; 4 Fujian Provincial Key Laboratory for Soft Functional Materials Research, Si Ming Nan Lu 422, 361005 Xiamen, China; 5 Material Physics, BASF SE, Carl-Bosch-Straße 38, 67056 Ludwigshafen, Germany; 6 Analytical Chemistry, University of Konstanz, Universitätsstraße 10, 78457 Konstanz, Germany

## Abstract

A key requirement for the understanding of crystal growth is to detect how new layers form and grow at the nanoscale. Multistage crystallization pathways involving liquid-like, amorphous or metastable crystalline precursors have been predicted by theoretical work and have been observed experimentally. Nevertheless, there is no clear evidence that any of these precursors can also be relevant for the growth of crystals of organic compounds. Herein, we present a new growth mode for crystals of DL-glutamic acid monohydrate that proceeds through the attachment of preformed nanoscopic species from solution, their subsequent decrease in height at the surface and final transformation into crystalline 2D nuclei that eventually build new molecular layers by further monomer incorporation. This alternative mechanism provides a direct proof for the existence of multistage pathways in the crystallization of molecular compounds and the relevance of precursor units larger than the monomeric constituents in the actual stage of growth.

## Abstract (web summary)

Multistage crystallization pathways involving nanoscopic precursors or intermediates have been proposed for various systems. Here, the authors find compelling evidence that nanoscopic species participate in the crystallization of glutamic acid monohydrate, extending this non-classical growth mechanism to organic crystals.

A growing body of evidence from the analysis of biominerals^1^,^2^, the crystallization of ionic compounds^3^,^4^,^5^,^6^ and proteins^7^,^8^,^9^,^10^, colloidal assembly^11^,^12^ and nanocrystal synthesis^13^,^14^,^15^ suggests that there are alternative routes^16^ to the formation of a crystalline solid that differ substantially from the picture drawn by classical nucleation theory^17^ and layer-by-layer growth models^18^. The fast development of high-resolution analytical techniques such as cryo-transmission electron microscopy^19^,^20^, *in situ* atomic force microscopy (AFM)^7^,^10^ and liquid-cell transmission electron microscopy^15^ now enables the detection of precursors in their native state during the early stages of the precipitation of atomic, ionic or molecular compounds^21^. In particular, AFM has been successfully applied to observe classical layer-by-layer growth of proteins in solution^22^ as well as multilayer crystallization via liquid droplet precursors^7^,^10^,^23^,^24^,^25^, where the liquid-like state of surface-attached precursor species could be experimentally verified^26^. For inorganic compounds, recent theoretical^27^,^28^ and experimental work^5^,^29^,^30^,^31^ has indicated the possibility of the existence of complex nonclassical mineralization pathways that can involve prenucleation clusters (PNCs)^32^ and hydrated nanodroplets that form from the PNC precursors at some critical concentration upon liquid–liquid demixing^33^. Subsequently, driven by the reduction of interfacial surface area, the nanodroplets aggregate and/or grow by inclusion of monomers to yield larger solvent-rich mineral droplets that later dehydrate into amorphous nanoparticles^33^ and finally crystallize^31^. While this pathway envisages a vital role of PNCs and/or nanoscopic liquid intermediates in the process of phase separation, it does not (yet) consider their possible relevance during the later stages of growth. Recently, Lupulescu and Rimer^34^ reported that nanoscopic species (significantly larger than monomeric building units) are involved in the growth of inorganic silicalite-1 crystals. However, such crucial evidence is still missing for the crystallization of small organic molecules.

In the present work, we address this issue for the case of DL-glutamic acid (DL-Glu). Arguably, a prerequisite for any nonclassical crystal growth modes is the existence of nanoscopic species in solution. This is explored by three independent methods: electrospray ionization mass spectrometry (ESI-MS), analytical ultracentrifugation (AUC) and *in situ* AFM on an inert silicon substrate.

## Results

### Electrospray ionization mass spectrometry

**Figure 1:**
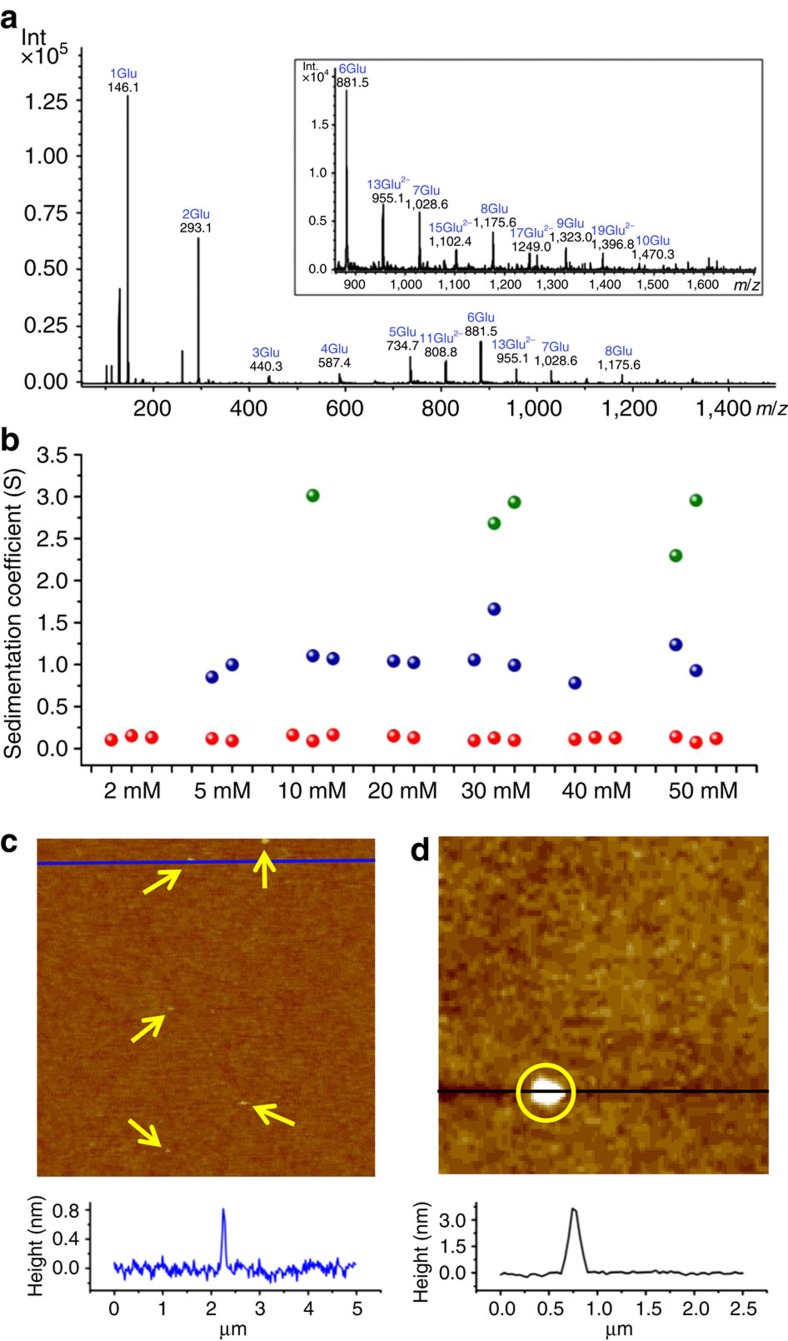
Evidences of different cluster species by using different analytical tools. (**a**) ESI Ion-Trap mass spectrum (negative ion mode) of a 40 mM solution of DL-Glu in water at pH 11 (adjusted with NH_4_OH). Inset: zoom into the high *m/z* range. (**b**) Sedimentation coefficients of species detected by AUC at 60,000 r.p.m. in D_2_O solutions of DL-Glu at different concentrations, as resulting from two or three independent experiments (see Supplementary Fig. 3 for corresponding results in H_2_O). (**c**,**d**) The *in situ* AFM images of species deposited from a supersaturated solution of DL-Glu on the (100) surface of a silicon wafer. The plots at the bottom are height profiles along the blue and black lines in the images. Fields of view: 5 μm in **c** and 2.5 μm in **d**.

ESI-MS is a soft ionization method that is capable of preserving even weak intermolecular interactions, allowing the transfer of non-covalent complexes of a wide range of (bio)molecules from solution into the gas phase, where they can be detected by MS analysis, as shown previously for amino acids and other small molecules^35^, as well as polypeptides and even large biopolymers. However, it should be noted that the probability of solution species to become successfully transferred into the gas phase decreases with their molecular weight. Therefore, ESI-MS serves primarily to explore the range of species present, while it cannot provide a quantitative description of their relative abundance. Corresponding spectra of DL-Glu in water display a broad spectrum of cluster species with different sizes and charges in both positive and negative ion modes (Fig. 1a). The particular size distribution depends on parameters like the amino acid concentration, pH, salt content and co-solvent, but the data clearly demonstrate the existence of fairly large clusters in solution under all investigated conditions. The largest cluster observed in 40 mM solutions of DL-Glu was a doubly charged 19-mer at pH 11 in the negative ion mode (see Supplementary Fig. 1 for data at the native pH of 3.2). Interestingly, analyses of peak intensities in the mass spectra show a clear increase in the fraction of larger oligomeric species with increasing DL-Glu concentration in the undersaturated regime (see Supplementary Fig. 2), suggesting that even larger entities will occur beyond the saturation limit (ca. 82 mM, as determined gravimetrically). Again, we note that average cluster sizes determined in this way do not necessarily reflect the true situation in solution due to the lower probability for ionization of larger species. In any case, it is remarkable that ESI-MS detects large clusters in undersaturated solutions^35^. In principle, these species could be metastable precritical clusters populating local minima in the free energy landscape (that is, Δ*G*_cluster_>0)^36^, or thermodynamically stable PNCs (for which Δ*G*_cluster_<0), and the ESI-MS data alone do not allow distinguishing the two cases.

### Analytical ultracentrifugation

To further explore the existence of clusters in DL-Glu solutions and to investigate their thermodynamic stability, AUC experiments were performed at different concentrations, angular velocities and employing different solvents (H_2_O versus D_2_O). The results confirm the presence of clusters in the undersaturated regime and show up to three distinct components (see Fig. 1b and Supplementary Fig. 3): amino acid monomers at sedimentation coefficients of 0.1–0.2 S (corresponding to hydrodynamic diameters (*d*_H_) of 0.6–0.7 nm), a first population of clusters at 0.8–1.5 S (1.6≤*d*_H_≤2.5 nm) and occasionally another still larger species at 2–4 S (3<*d*_H_<4 nm). Evaluation of the AUC data also yields the relative concentrations of the different populations, and where detected, the first cluster population (0.8–1.5 S, Fig. 1b) constitutes a fraction of ca. 0.01–0.1% (w/w) relative to the monomers (0.1–0.2 S), consistent with previous AUC analyses on other amino acids^35^. However, the error underlying this determination is relatively large and thus no clear trend for any dependence of the cluster fraction on the DL-Glu concentration can be inferred. Nevertheless, the data allow assessing the sign of Δ*G*_cluster_ (ref. 37) if the number of glutamic acid monomers in the clusters is known. An estimate for the degree of association can be obtained by combining sedimentation coefficients with the simultaneously measured diffusion coefficients (Svedberg equation; see Supplementary Note 1), yielding typical numbers of 15–25 Glu monomers in the species with *s*≈1 S. Indeed, clusters of this size were detected independently by ESI-MS (cf. Fig. 1a), and considering the molecular weight dependence of detection in the gas phase as discussed above^38^, it seems reasonable to assume that aggregates comprising ∼20 glutamic acid molecules, on a global average in solution, provide a good representation of the species observed by AUC. It is noteworthy that AUC is an absolute technique that detects all existing species on a statistically relevant basis, as opposed to ESI-MS.

The equilibrium for the formation of a cluster consisting of 20 Glu monomers can be formally described by the equilibrium constant *K*_cluster_=[Glu_20_] [Glu]^−20^ (note that this does not mean to imply a one-step association into one defined cluster species). For thermodynamically stable clusters (that is, Δ*G*_cluster_<0), the equilibrium constant is larger than unity, that is, *K*_cluster_>1 (with Δ*G*_cluster_=−*RT* ln*K*_cluster_). For DL-Glu concentrations of 5 and 50 mM (cf. Fig. 1b), this would be the case for [Glu_20_] concentrations down to as low as ∼10^−46^ M and ∼10^−26^ M, respectively. Even if the first cluster population was merely a pentamer on average, *K*_cluster_ would still be larger than unity for detected cluster concentrations larger by ∼0.3 μM (that is, >ca.0.003% relative to the monomers) in 50 mM DL-Glu solutions. This nicely illustrates that even though the population of amino acid clusters is relatively small and constitutes fractions of only 0.01–0.1% (w/w) relative to the monomers in solution, it still can represent a thermodynamically stable state commensurate with the definition of PNCs.

The fact that AUC (as a slow experiment) can resolve two or three cluster populations next to free monomers suggests that these species are not linked by fast equilibria, as found for calcium carbonate^3^,^39^. This indicates that at least a fraction of the clusters observed in the present work are nanodroplets with a phase boundary that may result from liquid–liquid demixing^32^. While the liquid-like character of the species observed in this work remains hypothetical, there is evidence that fairly large entities can exist already in undersaturated solutions of amino acids^40^,^41^. Indeed, the dynamics of organic clusters might generally be slower than those of inorganic compounds, and a qualitative assessment of cluster dynamics by means of AUC cannot provide unambiguous evidence about whether or not the detected clusters are actually phase separated. Also, the much larger amino acid associates reported by Hagmeyer *et al*.^40^ and Jawor-Baczynska *et al*.^41^ could actually constitute a mesophase that is part of the homogeneous solution and thereby does not violate Gibbs’ phase rule^42^. In any case, density-variation AUC experiments show that the mole fraction of water in the clusters is ca. 0.34 (see Supplementary Note 2), demonstrating that the nanospecies in solution are highly hydrated in nature.

### Atomic force microscopy

To further confirm the existence of nanoscopic entities in solution, we conducted an *in situ* AFM experiment using silicon wafers as an inert substrate. In this case, two populations of nanosized species were observed at the surface (Fig. 1c,d and Supplementary Fig. 4). On the one hand, we detected very small clusters (marked by arrows in Fig. 1c) that attached transiently to the surface and had disappeared already in the next frame of the same area. Their measured height of ca. 1 nm is commensurate with the smaller populations detected by ESI-MS and AUC, and agrees with the size reported for PNCs of both ionic minerals^3^ and amino acids^35^ in the literature. Whether the fast disappearance of these species is due to Brownian motion, dissociation into small fragments or coalescence into larger entities cannot be inferred from the present data. On the other hand, we also observed distinctly larger structures, initially ∼2–4 nm high and 150–300 nm wide, attaching to the silicon surface (Fig. 1c,d and Supplementary Fig. 4a) and decreasing in height with time (see Supplementary Fig. 4b). These species may correspond to the mesoscopic aggregates reported in the literature and are clearly too large to be detected by AUC and ESI-MS.

**Figure 2:**
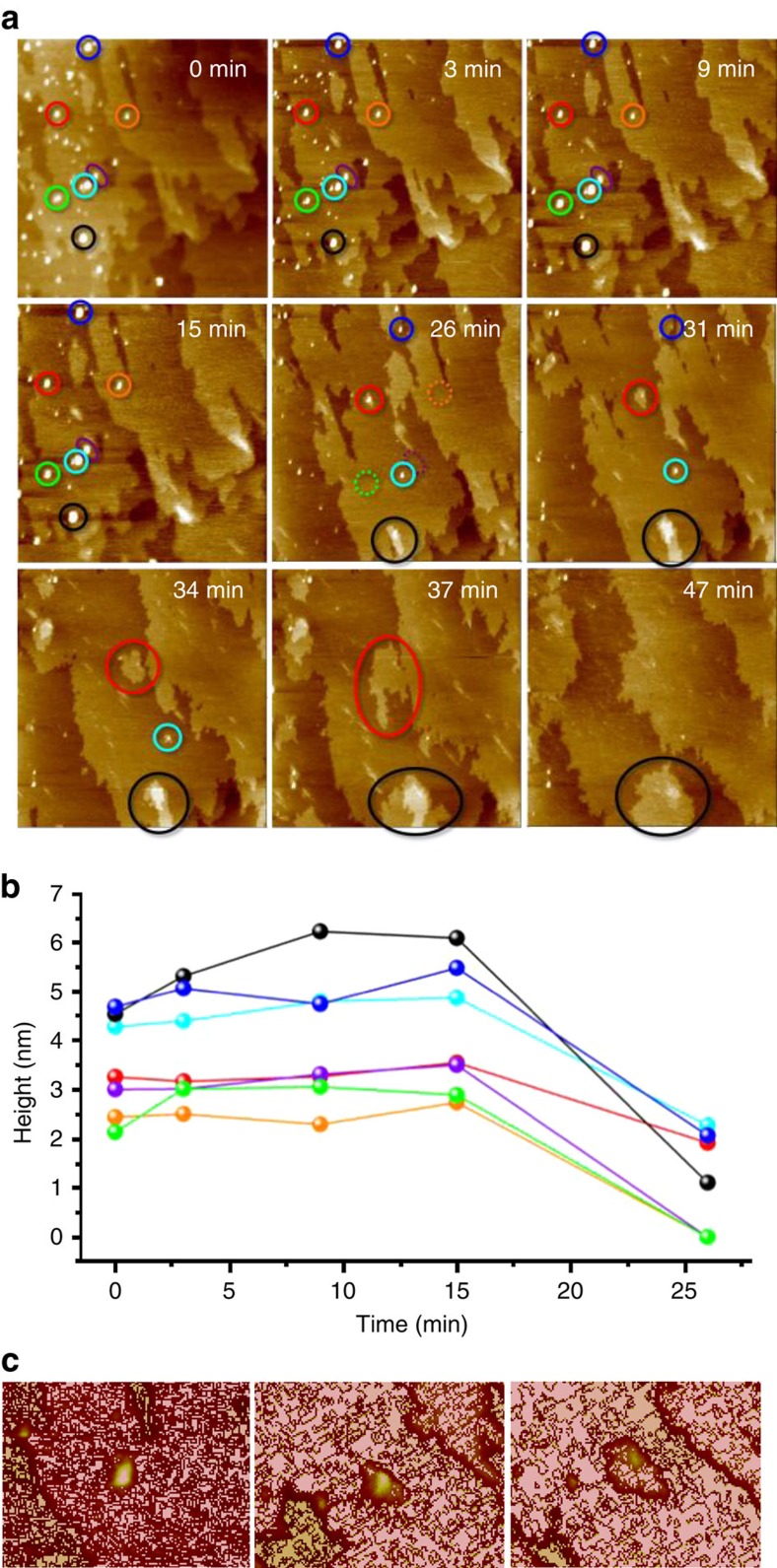
Growth of crystals via the attachment and relaxation of preformed nanospecies. (**a**) Time-lapse sequence of representative AFM images showing the deposition and subsequent morphological change of the precursors on a preexisting {110} surface of a Glu·H_2_O single crystal. The first image (*t*=0 min) was taken ∼5 min after addition of the supersaturated solution. Field of view: 2 μm. (**b**) Height change as a function of time for seven selected nanodroplets marked with coloured circles in the images shown in **a**. Note that molecular layers of ca. 0.4 nm in height formed around the nanospecies as they decreased in height over time. (**c**) From left to right, close-up AFM views of the nanodroplet marked in red in **a** at 15, 26 and 31 min, showing the transformation of the attached nanospecies into a new layer at the surface. Field of view: 500 nm.

To investigate the role of the detected nanoscopic entities during actual crystal growth, we performed *in situ* AFM studies on the {110} facets of a Glu·H_2_O crystal that was immersed in a supersaturated mother solution. Figure 2a shows a series of nine AFM frames collected at different times, where the attachment of nanosized species from solution on the preexisting crystal substrate and their subsequent evolution at the surface are clearly visible. Time-dependent changes in height of the seven highlighted nanoscopic entities are summarized in Fig. 2b. At the beginning, all deposited species exist as three-dimensional (3D) structures on the parent crystal surfaces with heights ranging from 2 to 5 nm (and diameters of 20–80 nm)—obviously much larger than a single molecular layer of Glu·H_2_O (ca. 0.4 nm, as estimated from the structure of the {220} facet, see Supplementary Fig. 5) and initially similar to the larger structures observed on the inert silicon substrate (Fig. 1d). In the following 15 min, a slight increase in height can be discerned for some of the surface-attached nanoscopic entities, likely due to the inclusion of further amino acid molecules from solution—either as monomers or in the form of larger associated species (that is, PNCs or primary nanodroplets). Starting from ∼15 min, a significant decrease in height is observed for most of the deposited species, resulting in the disappearance of some of the smaller particles in the frame taken after 26 min (highlighted by green, purple and orange circles in Fig. 2a). This is particularly evident when comparing, for example, the features highlighted by red and purple circles in Fig. 2a that have similar initial heights but develop along different pathways: the red one persists for the subsequent two-dimensional (2D) growth while the purple one redissolves, possibly by direct mass transfer to the adjacent precursor marked by the cyan circle. Intriguingly, the remaining nuclei start to grow laterally into single new crystalline layers with the same height as those in the parent crystal (that is, ca. 0.4 nm), as illustrated by the magnified sequence in Fig. 2c. Thus, 2D nuclei for new molecular layers can form via structural relaxation (accompanied by a decrease in height) of preformed 3D nanospecies, acting as an initial material depot for subsequent epitaxial growth. By measuring the areas of newly grown layers in the AFM height images taken from 26 to 47 min, the fraction of the increased surface resulting from the attachment and transformation of nanoscopic entities from solution is ∼20% (see Supplementary Note 3). This may suggest that monomer addition from solution dominates crystal growth, although any role of PNCs, nanodroplets or nanoparticles in the growth of the remaining 80% of monolayers cannot be categorically ruled out. Indeed, on silicon, the surface-attached nanoscopic species do not transform into 2D monolayers after their height has decreased to ca. 1–2 nm (cf. Fig. 1c,d and Supplementary Fig. 4). This demonstrates that the preexisting crystalline Glu·H_2_O surface plays a crucial role in decreasing the barrier to epitaxial growth via heterogeneous nucleation. The silicon surface, in turn, lacks enough structural similarity to promote the formation of 2D nuclei due to lattice mismatch, as discussed previously to explain the failure of S-layer protein assemblies to grow on a bare silicon substrate^10^.

The findings presented above differ fundamentally from the results of previous AFM studies on the crystallization of both ionic minerals and proteins. The classical scenario of mineral growth assumes that new layers are initiated by the direct formation of crystalline 2D nuclei^18^. Such a ‘birth and spread’ pathway has been evidenced for the growth of calcite^43^ and other minerals. In turn, 3D nuclei in protein crystallization—which are assumed to originate from liquid–liquid demixing^9^—transform directly into crystalline multilayers with heights similar to that of the initial precursor^7^, rather than spreading out into one single layer as observed in the present work. The data in Fig. 2 indicate that crystallinity emerges only during the decrease in height as amorphous 3D nanoscopic entities transform into crystalline 2D layers^12^. In the present case, we hypothesize that the release of water from hydrated disordered precursors back into the surrounding solution is entropically favourable, and thus drives the assembly of remaining solute molecules into an ordered structure on the underlying parent crystal substrate^44^.

## Discussion

We note that the precursor structures observed by AFM at both the growing Glu·H_2_O surface and the inert silicon substrate (several nm in height, but up to 300 nm in width upon partial wetting) are distinctly larger (with respect to volume) than the species detected by both ESI-MS and AUC in solution (<5 nm in diameter). This may indicate that the solute entities traced by ESI-MS and AUC in the undersaturated regime (that is, PNCs and/or small droplets) undergo a further step of monomer attachment and/or aggregation and coalescence at higher concentrations^3^,^4^,^32^, yielding larger nanospecies^28^ that then become active in the growth process. However, it is also possible that the used techniques are simply not capable of capturing the entire spectrum of aggregated species in the system under the given conditions, as already mentioned above. In ESI-MS, the probability of successful transfer into the gas phase is known to decrease with increasing size of the oligomeric entities. Accordingly, we found a saturation of the intensity of the largest species at ∼20 mM and could not detect any larger aggregates of Glu molecules in significant amounts at higher concentrations (cf. Supplementary Fig. 2). AUC, on the other hand, may suffer from the low number density of larger nanoscopic species, their relatively high sedimentation coefficients and diffusion broadening effects. Therefore, we performed AUC experiments at variable angular velocity to look for larger species in solution. Indeed, clusters with diameters of *d*_H_>4 nm (*s*≈5 S) were traced at intermediate centrifugal force (see Supplementary Fig. 6), confirming the notion that the various techniques applied in this work preferentially capture different populations within a broad distribution of nanoscopic species existing in the system.

**Figure 3:**
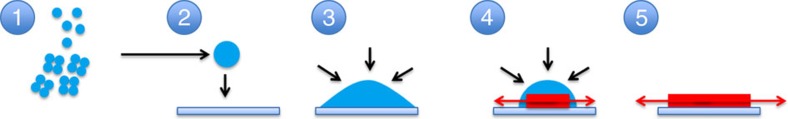
Scheme illustrating the multistage crystal growth pathway. We propose that PNCs (small blue dots, stage 1) undergo phase separation and aggregate to form larger nanodroplets (large blue sphere, stage 2) that attach to the crystal surface and spread to lower the interfacial energy (stage 3). Transformation of the 3D nanoscopic precursor (stage 4) leads to 2D nuclei that then grow into a new molecular layer nourished by the nanospecies that gradually decrease in height and ultimately disappear completely (stage 5).

In summary, our results demonstrate that growth of organic crystals can be a complex process with multiple stages that may involve PNCs, liquid nanodroplets or nanoparticles as precursors and/or growth units, as shown schematically in Fig. 3.

This study has shown that disordered nanoscopic precursors are not only relevant for the nucleation of crystals, but can also play an active role in the later stage of growth, where they serve as material depot and thus constitute an important alternative to classical molecule-by-molecule attachment. Evidence collected here and in previous work^10^ indicates that structural relaxation is the rate-determining step in this growth mechanism. Multistage pathways including PNCs, liquid nanodroplets or nanoparticles challenge our current understanding of crystallization, but clearly they also offer previously unrecognized handles for controlling such processes.

## Methods

### Sample preparation

DL-glutamic acid monohydrate was obtained as a powder from Sigma-Aldrich and used as received. For the AFM experiments, saturated solutions of DL-Glu·H_2_O were prepared by dissolution of the powder in water at 50 °C. After cooling to 20 °C, the solution was filtered through membranes with a pore size of 0.45 μm, giving a metastable supersaturated mother liquor suitable for monitoring the growth of DL-Glu·H_2_O by AFM. The initial level of supersaturation was *S*_50 °C_/*S*_20 °C_=2.1 (where *S*_*i*_ is the solubility of DL-Glu at the respective temperature). There was no bulk precipitation from this supersaturated solution during the AFM measurements. Samples for ESI-MS and AUC were obtained by dissolving the appropriate amount of DL-Glu·H_2_O in water at room temperature. In some cases, the pH was adjusted by addition of aliquots of NH_4_OH, NaOH or Ca(OH)_2_ solutions. All solutions were prepared with water of Milli-Q quality.

### Atomic force microscopy

The *in situ* AFM measurements were performed using a fluid cell mounted on a multimode atomic force microscope from Veeco Instruments that was equipped with a liquid-resistant vertical engagement scanner (AS-12V) and operated in tapping mode. The probe consisted of a sharp silicon tip (Arrow-NCR-W; thickness: 4.6 μm, length: 160 μm, width: 45 μm; resonance frequency: 285 kHz, force constant: 42 N m^−1^) attached to a silicon nitride cantilever. Scan frequencies were between 1 and 8 Hz. A minimum loading force of ∼150 pN or less was applied using the optimized feedback and set-point parameters for good imaging conditions. AFM images were analysed with the software Nanoscope III.

For measurement, a piece of clean Si wafer was fixed on the metal disk by epoxy glue. As a reference, scanning was performed directly on the silicon surface in air (Supplementary Fig. 7a), water (Supplementary Fig. 7b) or supersaturated DL-Glu mother solution (cf. Fig. 1c,d in the main text).

To monitor growth of DL-glutamic acid monohydrate, a single crystal of DL-Glu·H_2_O was glued on the Si wafer. Subsequently, ∼0.2 ml of a supersaturated aqueous solution of DL-Glu was pumped into the fluid cell. Both the crystal and the AFM tip were covered by the mother liquor. Scanning was then performed directly on the surface of the crystal.

### Electrospray ionization mass spectrometry

Mass spectra were acquired with an Esquire 3000+ Ion-Trap Mass Spectrometer (Bruker Daltonik) in positive and negative ion modes. Samples were introduced by a syringe pump at a flow rate of 5 μl s^−1^. Spectra were recorded by scanning from 120 to 2,500 *m/z*. The ion source parameters were: 10 psi nebulizer gas (nitrogen), 6 l min^−1^ drying gas (nitrogen) with a temperature of 200 °C and capillary voltage 3,500 V. The existence and stability of glutamic acid clusters were probed by several series of measurements with Glu solutions at different concentrations (0.1–50 mM), different pH values (native and pH 11), in the presence and absence of an internal standard (aspartic acid), with and without co-solvent (methanol) and after different ageing times. Generally, very similar observations were made under all investigated conditions, that is, large oligomers were detected in all samples. There was no effect of ageing time on the results of the ESI-MS measurements.

### Analytical ultracentrifugation

AUC was performed on an Optima XL-I ultracentrifuge (Beckman-Coulter) at 25 °C using Rayleigh interference optics and double-sector Ti cells (Nanolytics). Measurements were carried out with solutions of DL-Glu at different concentrations (2–50 mM) in either H_2_O or D_2_O as solvent. Higher concentrations could not be measured due to increasing interferences caused by convection. The angular velocity was varied from 30,000 to 60,000 r.p.m., the latter being the standard value for the analysis of nanosized cluster species^39^. The obtained data were evaluated using the software SEDFIT by fitting the determined concentration profiles to the Lamm equation with a non-interaction model that yields the sedimentation coefficient, diffusion coefficient and concentration of each of the species. Data were fitted assuming the presence of one, two, three or four different components, and the best fit was chosen.

### Data availability

The data that support the findings of this study are available from the corresponding author on reasonable request.

## Additional information

**How to cite this article:** Jiang, Y. *et al*. Growth of organic crystals via attachment and transformation of nanoscopic precursors. *Nat. Commun.* **8**, 15933 doi: 10.1038/ncomms15933 (2017).

**Publisher’s note**: Springer Nature remains neutral with regard to jurisdictional claims in published maps and institutional affiliations.

## Supplementary Material

Supplementary Information
