# Erratum: Growth of organic crystals via attachment and transformation of nanoscopic precursors

**DOI:** 10.1038/ncomms16132

**Published:** 2017-07-17

**Authors:** Yuan Jiang, Matthias Kellermeier, Denis Gebauer, Zihao Lu, Rose Rosenberg, Adrian Moise, Michael Przybylski, Helmut Cölfen

Nature Communications 8: Article number: 15933; DOI: 10.1038/ncomms15933 (2017); Published 06 21 2017; Updated 07 17 2017

The original version of this Article contained an error in the spelling of the author Denis Gebauer, which was incorrectly given as Denis Gebaue. This has now been corrected in both the PDF and HTML versions of the Article.

