# Supplementary material for: Growth of organic crystals via attachment and transformation of nanoscopic precursors
Source: Nat Commun. 2017 Jun 21;8:15933. doi: 10.1038/ncomms15933 (PMC5482053; doi:10.1038/ncomms15933)
Supplement: Supplementary Information [file ncomms15933-s1.pdf]

Type of file: PDF
Title of file for HTML: Supplementary Information
Description: Supplementary Figures, Supplementary Tables, Supplementary Notes and Supplementary References.

Supplementary Figures

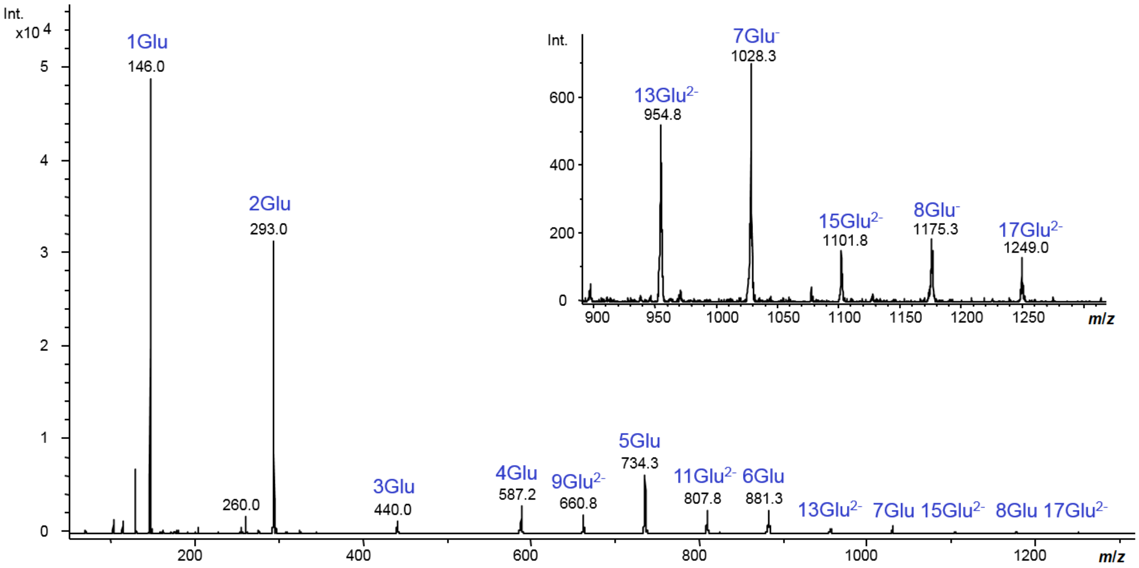

Supplementary Figure 1. Evidence of relatively large cluster species. ESI-IonTrap mass spectrum (negative ion mode) of a 40 mM solution of DL-Glu in water at the native pH of 3.2, showing the presence of clusters containing up to 17 monomers. Inset: zoom into the high *m/z* range.

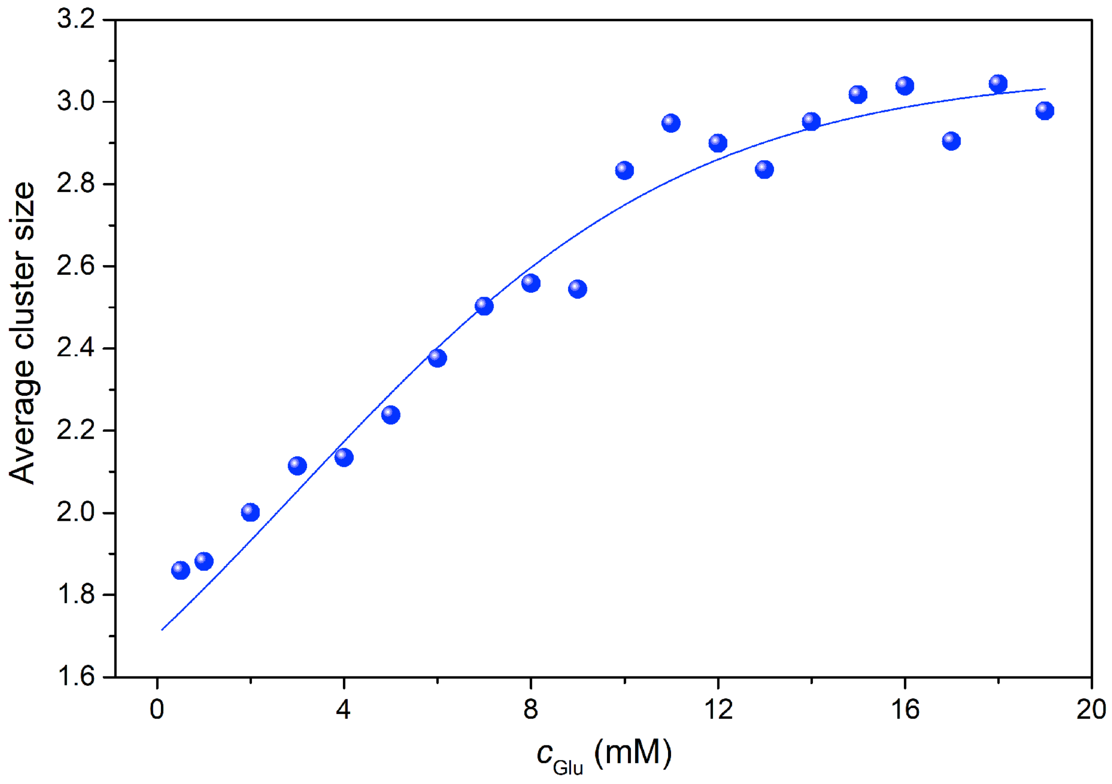

Supplementary Figure 2. Cluster size dependence on the solution concentration. Average cluster size (ACS) observed by ESI-MS analysis of aqueous DL-Glu solutions at different concentrations and the respective native pH (3-3.5). The data were derived from the peak intensities I_n of clusters consisting of n monomers via the following equation: ACS = $(\Sigma\ n \cdot I_n) / (\Sigma\ I_n)$[S1]. Note that the fraction of larger oligomers becomes more and more pronounced already at concentrations far below the solubility limit (ca. 82 mM as determined gravimetrically at the native pH). At concentrations higher than 20 mM, meaningful ESI-MS measurements were no longer possible due to saturation effects.

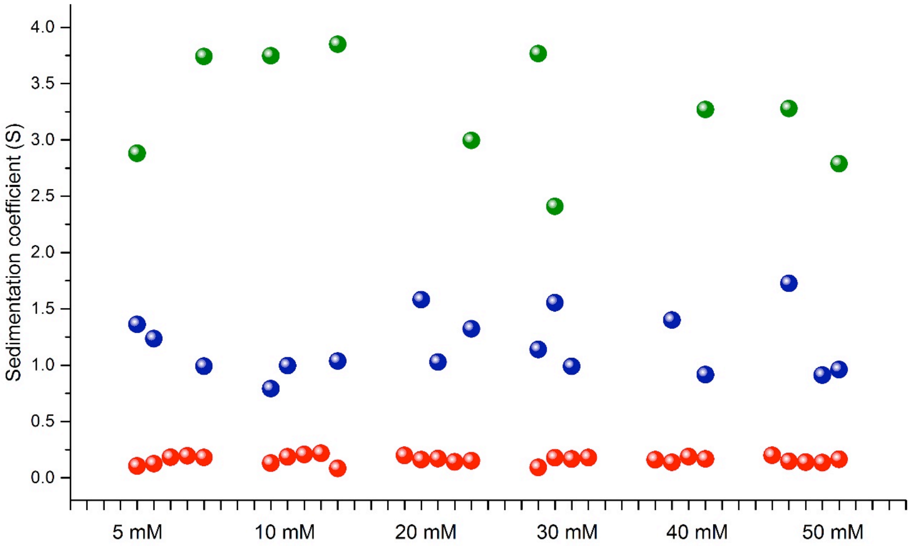

Supplementary Figure 3. Sedimentation coefficient dependence on the solution concentration. Sedimentation coefficients of species detected by AUC at 60,000 rpm in H_2O solutions of DL-Glu at different concentrations, as resulting from four or five independent experiments each. Data were evaluated assuming the presence of one, two or three components, and the best fit was chosen.

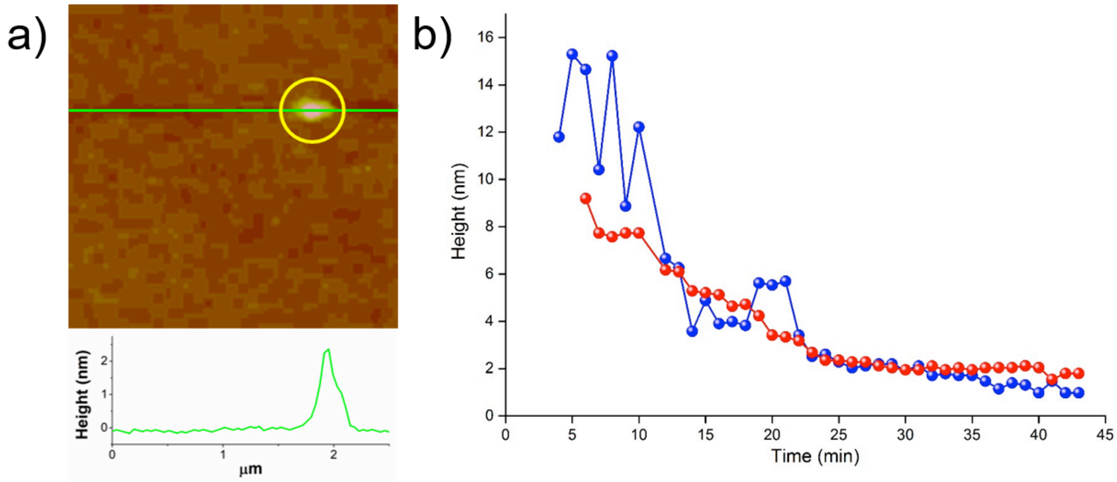

Supplementary Figure 4. Statistics of the height changes of clusters with time. a) Top: *In-situ* AFM image of one of the larger aggregates deposited from a supersaturated solution of DL-Glu on the (100) surface of a silicon wafer (field of view: 2.5 µm); bottom: height profile along the green line. b) Height change as a function of time for the large species highlighted by yellow circles in (a) (red curve) and Fig. 1d in the main text (blue curve). Note that the height of the observed entities decreases over time (likely due to progressive wetting of the substrate) and approaches a final level of 1-2 nm after about 30 min in both cases.

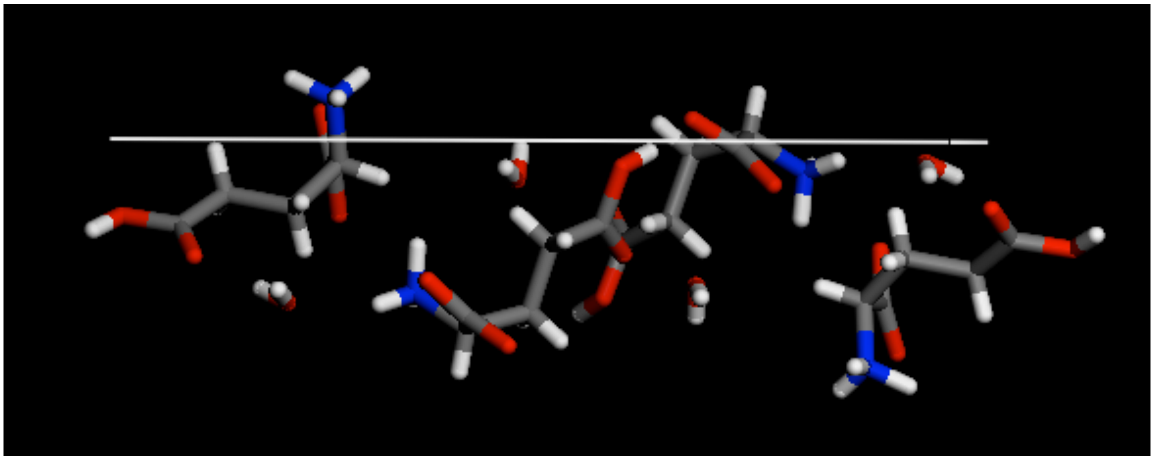

Supplementary Figure 5. Side view of the (220) facet of a DL-Glu·H_2O crystal. The *d*-spacing is 3.93 Å. The image was generated by Materials Studio 5.5 (Accelrys). Color code: gray = carbon, red = oxygen, blue = nitrogen, white = hydrogen.

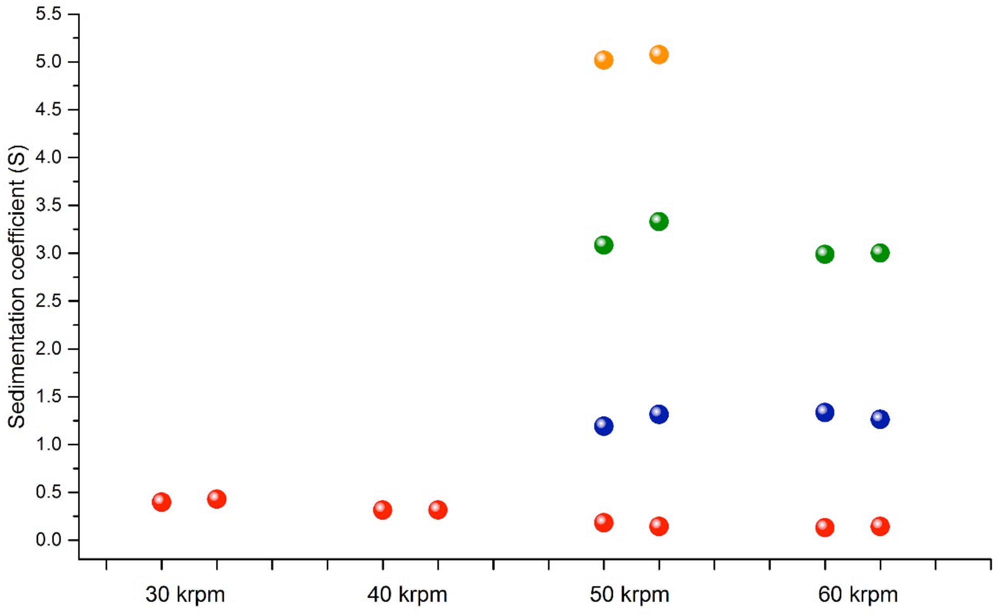

Supplementary Figure 6. Sedimentation coefficient dependence on the angular velocities. Sedimentation coefficients of species detected by AUC at different angular velocities in 50 mM solutions of DL-Glu in water, as resulting from two independent experiments each. Data were evaluated assuming the presence of one, two, three or four components, and the best fit was chosen. Note that an additional population at $s \approx 5$ S is observed at 50,000 rpm, which cannot be detected at the standard speed of 60,000 rpm due to too fast sedimentation. The absence of any associated species at 30,000 and 40,000 rpm can likely be ascribed to strong diffusion broadening effects, which limit the resolution of the technique under these conditions.

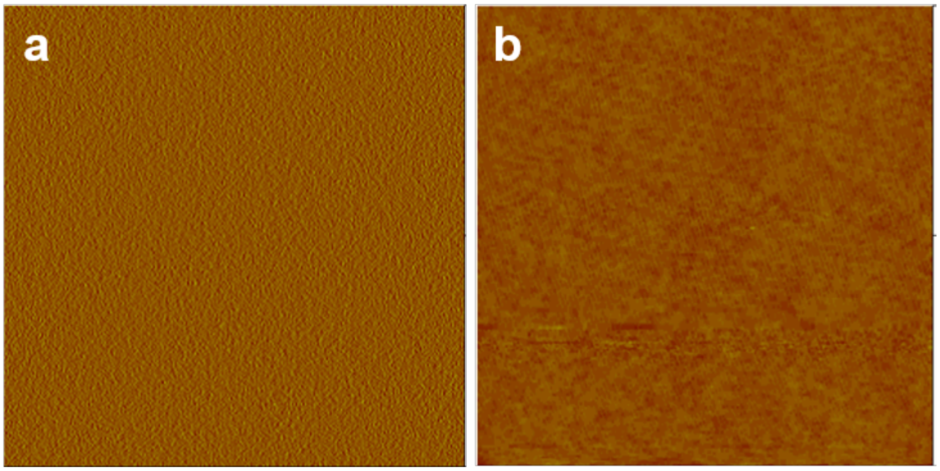

Supplementary Figure 7. Surface information of a bare silicon wafer. AFM images taken from the surface of a bare silicon wafer in a) air and b) water in the same area as shown for supersaturated DL-Glu solution in Fig. 1c-d of the main text. Note that there are no nanosized species on the surface in both cases. Field of view: 5 μm.

Supplementary Note 1

To estimate the molecular weight of the clusters/droplets and with it, the average number of monomers in these entities, we used the well-known Svedberg equation. Here, the sedimentation and diffusion coefficient (both measured simultaneously in the AUC experiment) as well as the partial specific volume are needed to calculate the molar mass according to:

$$M_{\text{Cluster}} = \frac{s \cdot R \cdot T}{D \cdot (1 - \bar{v}_{\text{Cluster}} \cdot \rho_{\text{H}_2\text{O}})} \quad (1)$$

The numbers of Glu monomers (N_{Glu}) and water molecules (N_{H2O}) are then accessible via:

$$M_{\text{Cluster}} = N_{\text{Glu}} \cdot M_{\text{Glu}} + N_{\text{H}_2\text{O}} \cdot M_{\text{H}_2\text{O}} = \frac{x_{\text{Glu}}}{x_{\text{H}_2\text{O}}} \cdot N_{\text{H}_2\text{O}} \cdot M_{\text{Glu}} + N_{\text{H}_2\text{O}} \cdot M_{\text{H}_2\text{O}} \quad (2)$$

Meaningful results were obtained for the samples at 20, 40 and 50 mM Glu, where the concentration is high enough to provide good signal and low enough to prevent ample convection. The supplementary table 1 gives the resulting composition of the clusters/droplets showing a sedimentation coefficient of $s \approx 1$ S (population 2):

Supplementary Table 1. Cluster composition dependence on the solution concentration.

c_{Glu} (mM)	M_{Cluster} (kDa)	N_{Glu}	N_{H2O}
**20**	3.26	21	11
**40**	3.67	23	12
**50**	2.49	16	8

Supplementary Note 2

The water content of the nanospecies was determined using AUC data obtained for the same sample measured in H_2O and D_2O (density variation experiment)[S3,S4]. This allows for the simultaneous calculation of the size and density when assuming that the clusters are spherical and that their degree of solvation is identical in both solvents, i.e. that no solvent exchange takes place. The latter assumption is particularly reasonable for nanodroplets, which form by liquid-liquid demixing and constitute a separate phase. Under these conditions, the density of the clusters/droplets (or respectively their partial specific volume) can be calculated from the measured sedimentation coefficients with the known viscosities and densities of the two solvents via the following equation:

$$\rho_{\mathrm{Cluster}} = \frac{s_{\mathrm{H_2O}} \cdot \eta_{\mathrm{H_2O}} \cdot \rho_{\mathrm{D_2O}} - s_{\mathrm{D_2O}} \cdot \eta_{\mathrm{D_2O}} \cdot \rho_{\mathrm{H_2O}}}{s_{\mathrm{H_2O}} \cdot \eta_{\mathrm{H_2O}} - s_{\mathrm{D_2O}} \cdot \eta_{\mathrm{D_2O}}} = \frac{1}{\bar{v}_{\mathrm{Cluster}}} \tag{3}$$

This gave ρ_{Cluster} = 1.484 g/mL (or a partial specific volume of 0.674 mL/g). The density is related to the composition according to:

$$\rho_{\mathrm{Cluster}} = w_{\mathrm{Glu}} \cdot \rho_{\mathrm{Glu}} + w_{\mathrm{H_2O}} \cdot \rho_{\mathrm{H_2O}} = (1 - w_{\mathrm{H_2O}}) \cdot \rho_{\mathrm{Glu}} + w_{\mathrm{H_2O}} \cdot \rho_{\mathrm{H_2O}} \tag{4}$$

where w is the corresponding mass fraction of water and Glu, respectively, in the clusters, and ρ_{Glu} is the inverse of the known partial specific volume of glutamic acid (0.658 mL/g)[S5]. Solving the above equation for $w(H_2O)$ and using the measured value for ρ_{Cluster}, we obtain $w(H_2O)$ = 0.06, which can be converted to a mole fraction of $x(H_2O)$ = 0.34 via:

$$x_{\mathrm{H_2O}} = \frac{1}{1 + \frac{M_{\mathrm{H_2O}}}{M_{\mathrm{Glu}}} \cdot \left(\frac{1}{w_{\mathrm{H_2O}}} - 1 \right)} \tag{5}$$

The hydrodynamic diameter (d_H) of the species detected in H_2O can now be calculated from the sedimentation coefficients and the cluster density determined as described above with the help of the following equation:

$$d_{\mathrm{H}} = \sqrt{\frac{18 \cdot \eta_{\mathrm{H_2O}} \cdot s}{\rho_{\mathrm{Cluster}} - \rho_{\mathrm{H_2O}}}} \tag{6}$$

Selected results obtained from a representative set of measurements in H_2O are given below:

Supplementary Table 2. Calculation of cluster sizes by using sedimentation coefficients.

c_{Glu} (mM)	s_1 (S)	s_2 (S)	s_3 (S)	$d_{H,2}$ (nm)	$d_{H,3}$ (nm)
**5**	0.181	0.990	3.741	1.85	3.60
**10**	0.084	1.034	3.849	1.90	3.66
**20**	0.152	1.323	2.995	2.14	3.22
**40**	0.168	0.916	3.269	1.78	3.37
**50**	0.164	0.961	2.788	1.83	3.11

Supplementary Note 3

In order to assess the relevance of the surface-attached nanoscopic species for the growth process of DL-Glu crystals as compared to competing growth mechanisms via monomer addition, we have measured the areas of newly formed layers in the AFM images at different times, distinguishing between domains that directly resulted from the transformation of nanospecies and layers that obviously formed without involving any detectable nanoscopic precursor. Supplementary Fig. 8 shows how the different regions were selected (Supplementary Fig. 8a , with red circles and colored polygons marking areas where growth occurred from nanoscopic precursors and smaller species from solution, respectively), and how the contour of a new layer was approximated (Supplementary Fig. 8b).

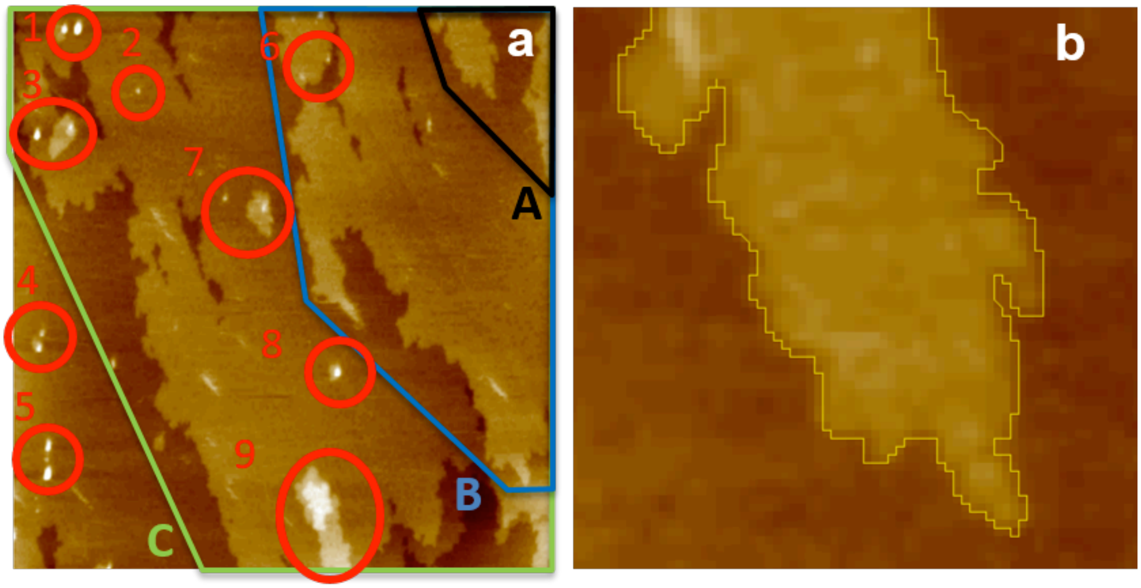

Supplementary Figure 8. **Determination of the crystal growth rates of new crystalline layers.** a) Exemplary AFM image (taken after 31 min, cf. Fig. 2a in the main text) showing the regions selected for calculating the areas of new layers formed by transformation of nanoscopic precursors (marked with red circles and numbered from 1-9) and addition of presumably monomeric units from solution (marked by colored polygons and labeled as A, B, and C). b) An exemplary image illustrating how the contour of a new layer was approximated. Fields of view: 2 µm (a) and 360 nm (b).

Every region was measured independently for three times using ImageJ, and the average value was taken as the final area. The supplementary table 3 gives the increase in area for new layers that were formed by monomer addition at step edges at different times from 26 to 47 min (in square pixels) as well as the percentage in the total area increase of new layers due to this growth mode.

Supplementary Table 3. Area increase of the new layer at different crystal growth periods.

Region	26 min	31 min	34 min	37 min	47 min
**A**	2159	3295	7136	9085	20025
**B**	38622	43268	48799	54337	75930
**C**	109756	118731	122680	134958	148855
**Sum**	150537	165294	178615	198380	244810
**Increase**		14757	13321	19765	46430
**Percentage**		85.4%	79.0%	61.4%	88.9%

Corresponding values for growth via transformation of surface-attached nanoscopic precursors are given below.

Supplementary Table 4. Statistics of the size changes of clusters at different crystal growth periods.

Region	26 min	31 min	34 min	37 min	47 min
**1**	302	295	973	1218	1052
**2**	29	37	93	650	134
**3**	167	588	1757	3266	10379
**4**	102	102	222	1213	1375
**5**	162	162	132	428	190
**6**	1487	1992	2328	2328	2328
**7**	261	528	1465	4451	4451
**8**	60	138	164	854	854
**9**	770	2024	2283	7436	6820
**Sum**	3340	5866	9417	21884	27683
**Increase**		2526	3551	12427	5799
**Percentage**		14.6%	21.0%	38.6%	11.1%

It is evident that growth via addition of monomeric units from solution accounts for 60-90% of the observed new layers, while the remaining fraction results from preformed nanoscopic entities that attach to the surface and transform into crystalline layers with time.

Supplementary References

1. Nemes, P., Schlosser, G. & Vekey, K. Amino acid cluster formation studied by electrospray ionization mass spectrometry. *J. Mass Spectrom.* **40**, 43-49 (2005)

2. Kellermeier, M., Cölfen, H. & Gebauer, D. in *Research Methods in Biomineralization Science* (Ed.: J. J. De Yoreo), *Methods in Enzymology* **532**, 45-69 (2013).

3. Edelstein, S. J. & Schachman, H. K. Simultaneous determination of partial specific volumes und molecular weights with microgram quantities. *J. Biol. Chem.* **242**, 306-311 (1967).

4. Mächtle, W. Characterization of dispersions using combined H_2O/D_2O ultracentrifuge experiments. *Macromol. Chem.* **185**, 1025-1039 (1984).

5. The program SDNTERP by John Philo was used for the calculation of the partial specific volume of glutamic acid. This program can be found using: http://www.jphilo.mailway.com/download.htm

   Going to the topic: "SEDNTERP-related files" this concerns Windows installations for different Windows versions. A Mac OS version can be found using: http://rasmb.org/sednterp/

   This information was collected on 13.5.2017
